# Supplementary material for: Comprehensive analysis of CXXX sequence space reveals that Saccharomyces cerevisiae GGTase-I mainly relies on a2X substrate determinants
Source: G3 (Bethesda). 2024 Jun 5;14(8):jkae121. doi: 10.1093/g3journal/jkae121 (PMC11304957; doi:10.1093/g3journal/jkae121)
Supplement: jkae121_Supplementary_Data [file jkae121_supplementary_data.zip › File_S2_G3-2024-404964.pdf]

Figure S1

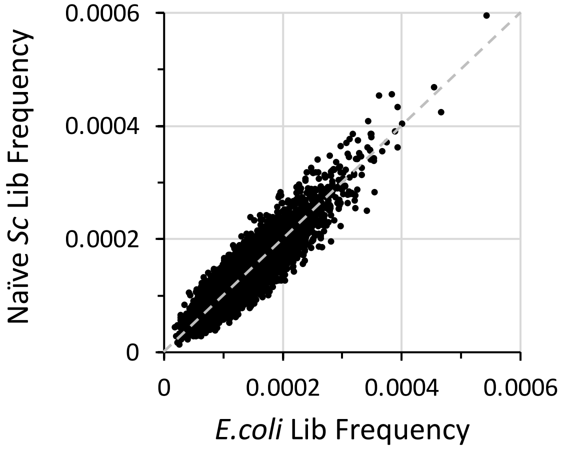

Figure S1. *Plot of frequencies observed for YDJ1-CXXX sequences in E. coli and naïve yeast libraries.* The frequencies of CXXX sequences within each library are unequal, yielding a range of frequencies. There is, however, a strong correlation (R^2 = 0.8271) between the frequency distributions observed in the two libraries, indicating no obvious enrichment or de-enrichment for specific YDJ1-CXXX sequences during the yeast transformation process.

Figure S2

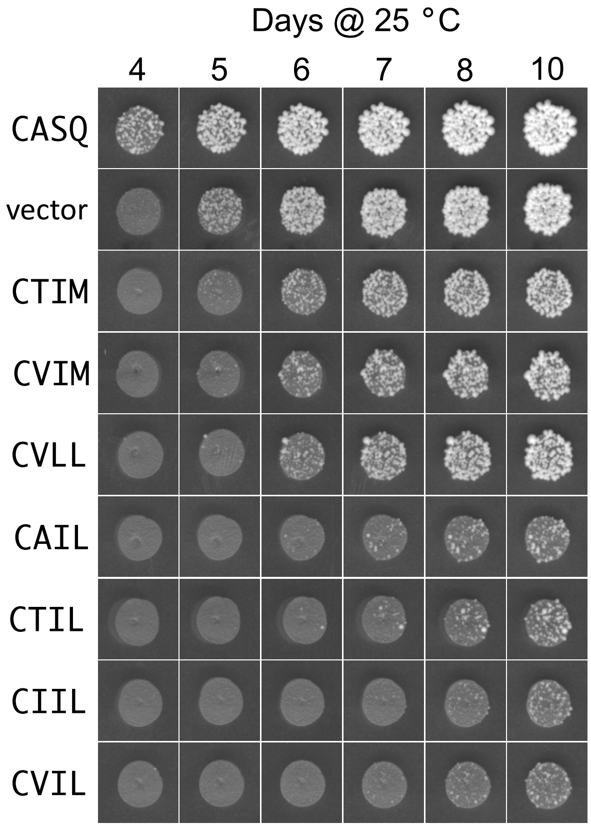

Figure S2. *Growth phenotypes of primary transformants expressing Ydj1-CXXX variants with geranylgeranylation potential.* Plasmids encoding the indicated Ydj1-CXXX variants were transformed in parallel into yeast lacking *RAM1* and *YDJ1* (yWS2542) using 1 µg of each plasmid. Transformed cells were gently harvested, resuspended to the same volume, and an equivalent portion of each transformation mixture manually spotted onto YPD solid media. Growth of colonies at room temperature was recorded over multiple days.

Figure S3

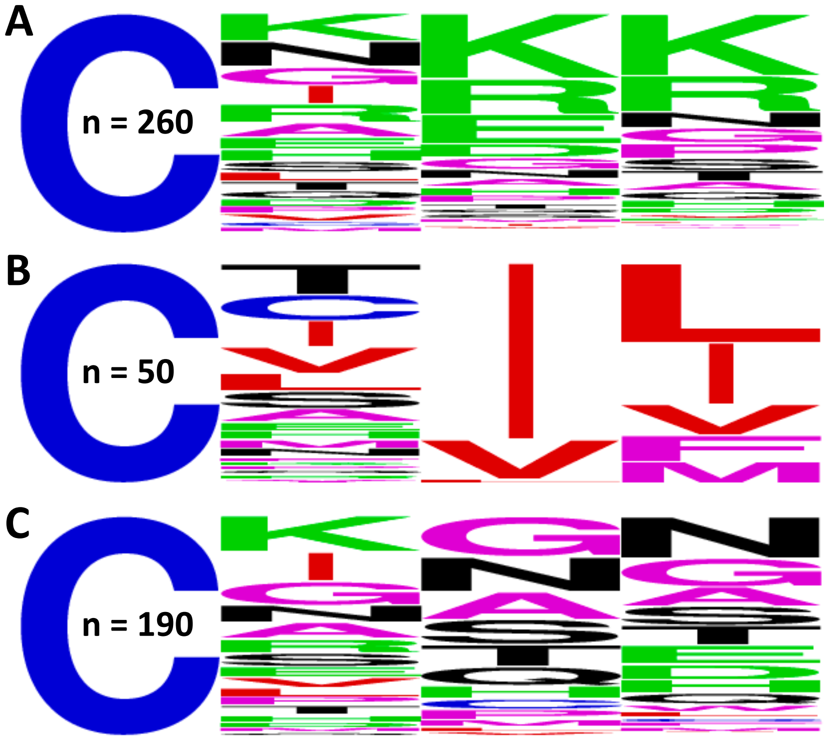

Figure S3. *WebLogo analyses of sequence subsets from the lowest 500 NGS E-Scores associated with the 42 °C data set.* The sequences evaluated **A**) have D/E/K/R at the a_2 position or K/P/R at the X position, **B**) match the consensus CX[V/I/L][L/F/I/M/V], or **C**) represent the remaining sequences after removing those evaluated in panels A and B.

Figure S4a

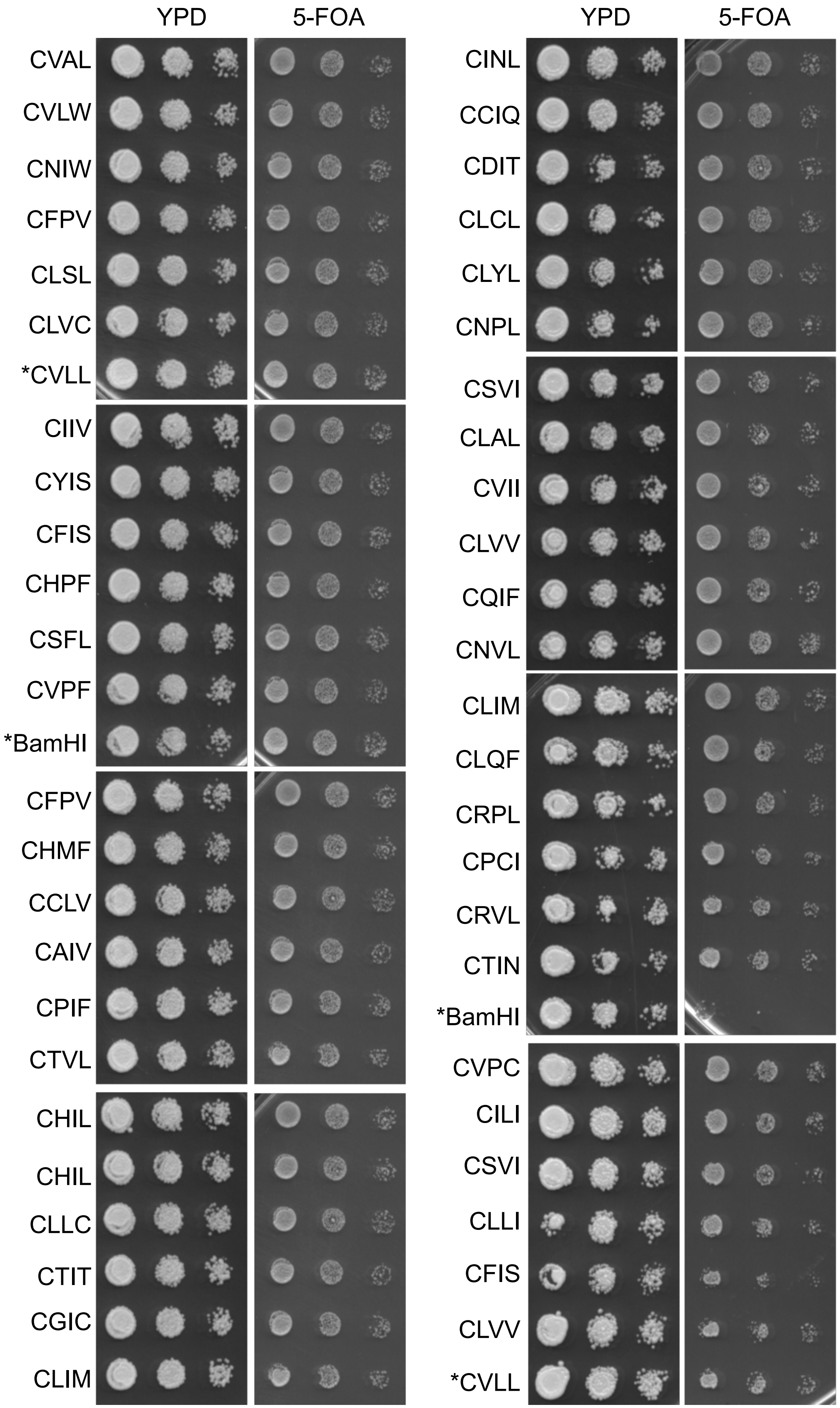
YPD
5-FOA
CVAL
CVLW
CNIW
CFPV
CLSL
CLVC
*CVLL
CIIV
CYIS
CFIS
CHPF
CSFL
CVPF
*BamHI
CFPV
CHMF
CCLV
CAIV
CPIF
CTVL
CHIL
CHIL
CLLC
CTIT
CGIC
CLIM
YPD
5-FOA
CINL
CCIQ
CDIT
CLCL
CLYL
CNPL
CSVI
CLAL
CVII
CLVV
CQIF
CNVL
CLIM
CLQF
CRPL
CPCI
CRVL
CTIN
*BamHI
CVPC
CILI
CSVI
CLLI
CFIS
CLVV
*CVLL

Figure S4b

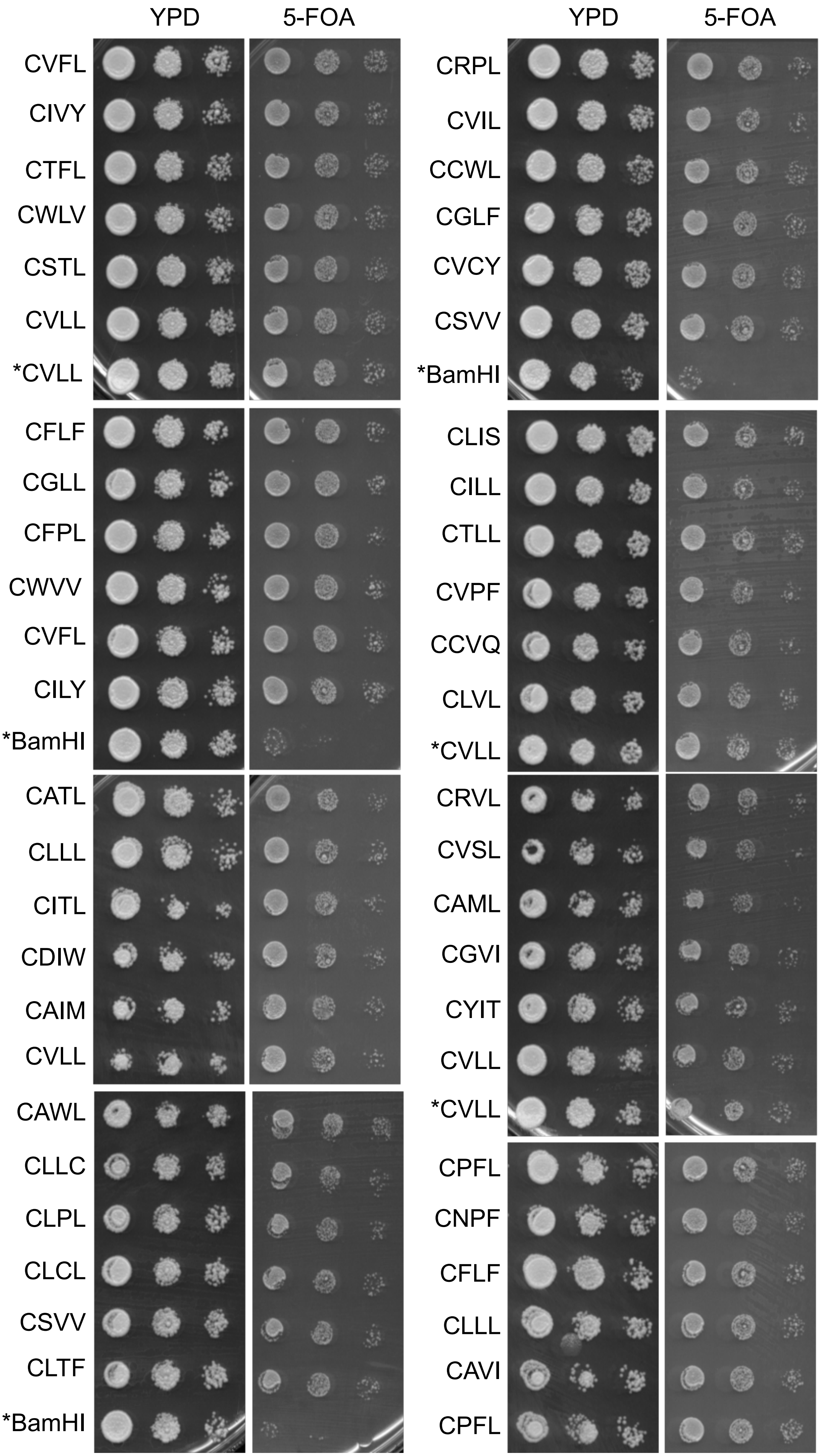

YPD
5-FOA
YPD
5-FOA
CVFL
CIVY
CTFL
CWLV
CSTL
CVLL
*CVLL
CRPL
CVIL
CCWL
CGLF
CVCY
CSVV
*BamHI
CFLF
CGLL
CFPL
CWVV
CVFL
CILY
*BamHI
CLIS
CILL
CTLL
CVPF
CCVQ
CLVL
*CVLL
CATL
CLLL
CITL
CDIW
CAIM
CVLL
CRVL
CVSL
CAML
CGVI
CYIT
CVLL
*CVLL
CAWL
CLLC
CLPL
CLCL
CSVV
CLTF
*BamHI
CPFL
CNPF
CFLF
CLLL
CAVI
CPFL

Figure S4c

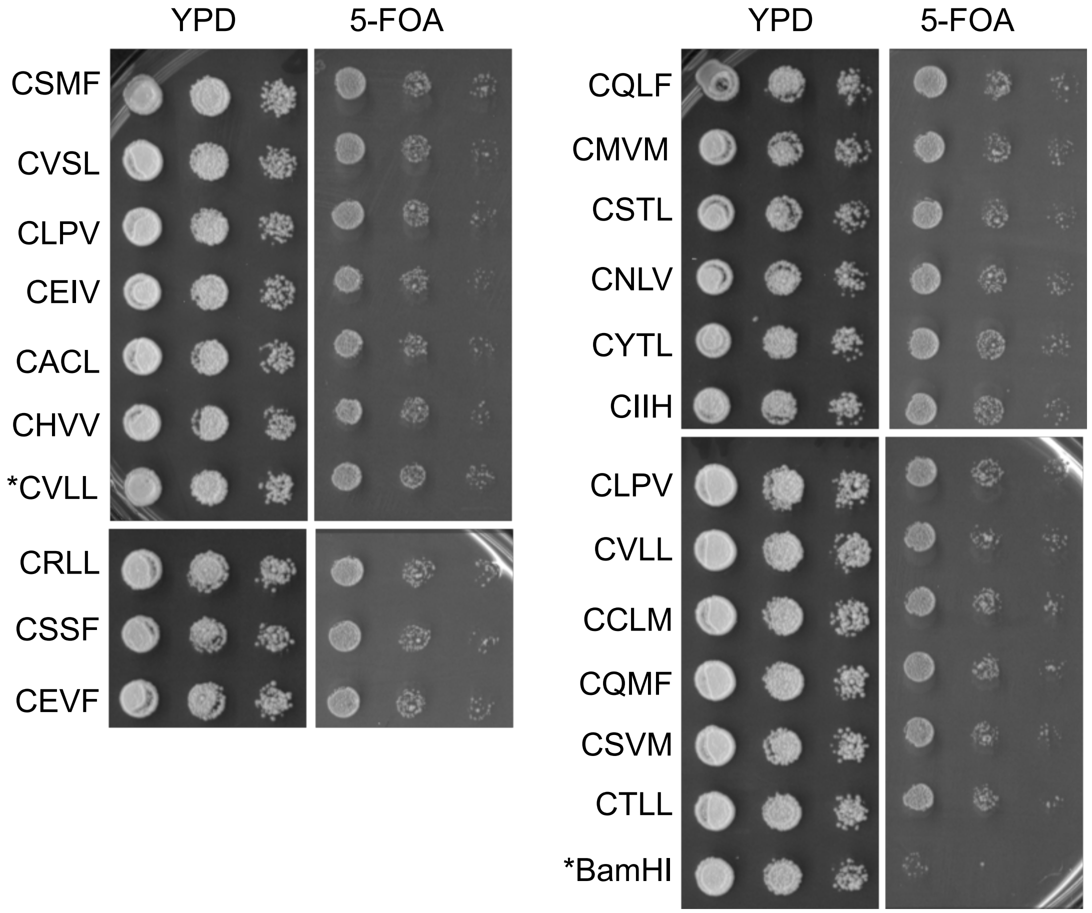

Figure S4. *Cell viability phenotypes of Rho1-CXXX variants from Rho1-based screen.* The 117 non-parent plasmids recovered by the strategy described in **Figure 7** were transformed individually into yWS3761 (*ram1Δ rho1Δ* [*CEN URA3 RHO1*]) and evaluated by the plasmid-loss assay. For each Rho1-CXXX variant, multiple colonies were used to inoculate SC-Leucine media. Saturated cultures were normalized to 1 A_{600} and used to prepare a 10-fold dilution series that was spotted onto YPD and 5-FOA media. The spotting on 5-FOA media plates was done in two technical replicates. The asterisk (*) denotes transformants expressing wildtype Rho1 (i.e., CVLL) or Rho1 lacking its entire CXXX sequence (i.e., BamHI) that were used as controls and evaluated multiple times.

Figure S5

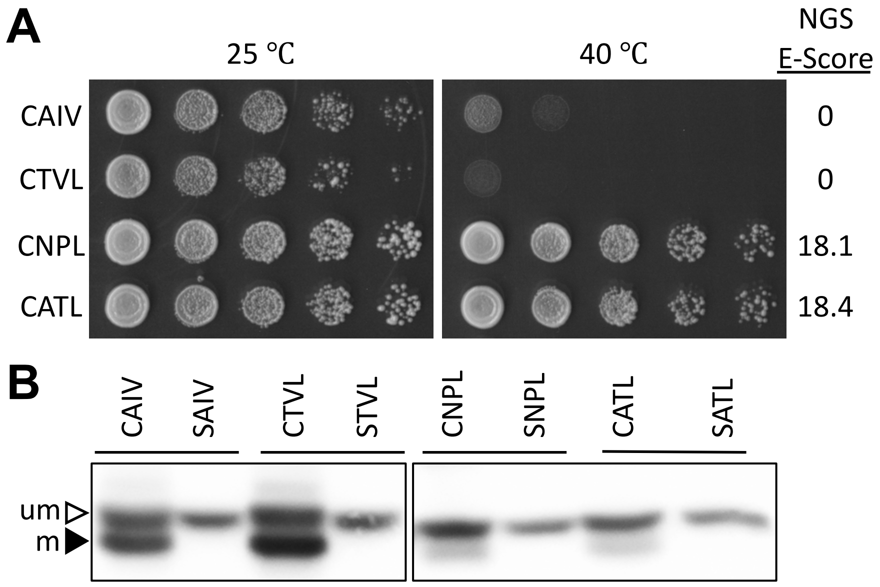

Figure S5. *Evaluation of Rho1-based CXXX hits in the context of Ydj1-based assays*. Plasmids encoding the indicated Ydj1-CXXX/SXXX variants were evaluated for **A**) thermotolerance and **B**) gel-shift as described in **Figures 1B and 5**, respectively. um – unmodified; m – modified. Data are representative of two biological replicates.

Table S1. Active site residues of β subunits in rat and yeast GGTase-I.

β subunit	a_2 specificity	X specificity	C20 specificity
*Rn*PGGT1B[a]	Thr49, Phe53, Leu320	Thr49, **His121[c]**, **Ala123**, Phe174	Thr49, Phe324
*Sc*Cdc43[b]	Trp108, Trp112, Tyr362	Trp108, **His156**, **Ala158**, Gly212	Trp108, Tyr366

[a]Previously reported (Taylor, Reid et al. 2003; Reid, Terry et al. 2004) or [b]determined by structure-based sequence alignment using the Align function of PyMol with the structures of *Rn*PGGT1B (PDB 1n4p) and *Sc*Cdc43 (AlphaFold) (Jumper, Evans et al. 2021). [c]Residues that are conserved based on structure-based sequence alignment are indicated in bold.

Table S2. Yeast plasmids used in this study.

Plasmid number	Genotype	Source
pRS315	*CEN LEU2*	(Sikorski and Hieter 1989)
pRS316	*CEN URA3*	(Sikorski and Hieter 1989)
pRS413	*CEN HIS3*	(Sikorski and Hieter 1989)
pRS425	*2µ LEU2*	(Sikorski and Hieter 1989)
pWS942	*CEN URA3 YDJ1*	(Hildebrandt, Cheng et al. 2016)
pWS1132	*CEN URA3 YDJ1-SASQ*	(Hildebrandt, Cheng et al. 2016)
pWS1321	*CEN URA3 YDJ1-CVLL*	(Hildebrandt, Sarkar et al. 2024)
pWS1461	*CEN URA3 YDJ1-CSFL*	(Berger, Yeung et al. 2022)
pWS1635	*CEN URA3 YDJ1-CVIL*	(Hildebrandt, Sarkar et al. 2024)
pWS1767	*CEN URA3 RAM1*	This study
pWS1775	*CEN URA3 YDJ1-CXXX*	(Kim, Hildebrandt et al. 2023)
pWS1807	*2µ LEU2 RAM1*	This study
pWS1835	*CEN URA3 RHO1*	This study
pWS1873	*CEN URA3 YDJ1-SSFL*	This study
pWS1885	*CEN LEU2* P_{PGK1}*-FNTA*	(Hildebrandt, Sarkar et al. 2024)
pWS1894	*CEN LEU2 RHO1*	This study
pWS1934	*CEN LEU2* P_{PGK1}*-PGGT1B*	(Hildebrandt, Sarkar et al. 2024)
pWS2101	*CEN LEU2 HA-RHO1*	This study
pWS2102	*CEN LEU2 HA-RHO1-CSFL*	This study
pWS2125	*CEN LEU2 HA-RHO1-BamH1*	This study
pWS2128	*CEN LEU2 RHO1-SVLL*	This study
pWS2129	*CEN LEU2 HA-RHO1-SVLL*	This study
pWS2133	*CEN URA3 YDJ1-CPLL*	This study
pWS2163	*CEN URA3 YDJ1-CWIT*	This study
pWS2164	*CEN URA3 YDJ1-CNTH*	This study
pWS2165	*CEN URA3 YDJ1-CETT*	This study
pWS2167	*CEN URA3 YDJ1-CDGE*	This study
pWS2174	*CEN URA3 YDJ1-CYVM*	This study
pWS2178	*CEN URA3 YDJ1-CVCG*	This study
pWS2197	*CEN LEU2 HA-RHO1-CASQ*	This study
pWS2202	*CEN URA3 YDJ1-CYIY*	This study
pWS2208	*CEN URA3 YDJ1-CLIN*	This study
pWS2225	*CEN LEU2 HA-RHO1-CVIL*	This study
pWS2234	*CEN URA3 YDJ1-SVLL*	This study
pWS2235	*CEN URA3 YDJ1-SVIL*	This study
pWS2237	*CEN URA3 YDJ1-SNTH*	This study

pWS2238	*CEN URA3 YDJ1-SLIN*	This study
pWS2241	*CEN LEU2 HA-RHO1-CYVM*	This study
pWS2243	*CEN LEU2 HA-RHO1-CWIT*	This study
pWS2244	*CEN LEU2 HA-RHO1-CVCG*	This study
pWS2247	*CEN LEU2 HA-RHO1-CNTH*	This study
pWS2249	*CEN LEU2 HA-RHO1-CLIN*	This study
pWS2253	*CEN LEU2 HA-RHO1-CETT*	This study
pWS2254	*CEN LEU2 HA-RHO1-CDGE*	This study
pWS2282	*CEN URA3 YDJ1-CAPL*	(Berger, Kim et al. 2018)
pWS2283	*CEN URA3 YDJ1-CRPL*	(Berger, Kim et al. 2018)
pWS2284	*CEN URA3 YDJ1-CFAL*	(Berger, Kim et al. 2018)
pWS2289	*CEN URA3 YDJ1-CAFL*	This study
pWS2290	*CEN URA3 YDJ1-CAIV*	This study
pWS2291	*CEN URA3 YDJ1-CATL*	This study
pWS2293	*CEN URA3 YDJ1-CNPL*	This study
pWS2294	*CEN URA3 YDJ1-CPIQ*	This study
pWS2297	*CEN URA3 YDJ1-CTVL*	This study
pWS2301	*CEN URA3 YDJ1-SAFL*	This study
pWS2302	*CEN URA3 YDJ1-SAIV*	This study
pWS2303	*CEN URA3 YDJ1-SATL*	This study
pWS2304	*CEN URA3 YDJ1-SNPL*	This study
pWS2305	*CEN URA3 YDJ1-SPIQ*	This study
pWS2306	*CEN URA3 YDJ1-STVL*	This study
pWS2307	*CEN LEU2 HA-RHO1-CPIQ*	This study
pWS2308	*CEN LEU2 HA-RHO1-CAFL*	This study
pWS2309	*CEN LEU2 HA-RHO1-CHLF*	This study
pWS2313	*CEN URA3 YDJ1-CHLF*	This study
pWS2326	*CEN LEU2* P_{PGK1}*-CDC43*	This study
pWS2327	*CEN LEU2* P_{PGK1}*-RAM2*	This study
pWS2350	*CEN HIS3* P_{PGK1}*-RAM2*	This study
SP319	*CEN URA3 HA-RHO1*	(Yoshida, Bartolini et al. 2009)

Table S3. Plasmid cloning strategies.

Plasmid number	Construction information	Vector backbone	Insert	Selection media
pWS1835	Recombination-mediated PCR-directed plasmid construction in yeast (BY4741)	Restriction enzyme digested linearized pRS316	PCR product encoding *RHO1* ORF	SC-Uracil
pWS1894	Recombination-mediated PCR-directed plasmid construction in yeast (BY4741)	Restriction enzyme digested linearized pRS315	*PvuI* digested 1835	SC-Leucine
pWS2101	Recombination-mediated PCR-directed plasmid construction in yeast (BY4741)	Restriction enzyme *digested* pWS1894	*PshAI* and *EcoRI* digested SP319	SC-Leucine
pWS2102	Recombination-mediated PCR-directed plasmid construction in yeast (BY4741)	Restriction enzyme digested pWS2101	PCR product encoding C-terminus of *RHO1* with the CXXX sequence altered to CSFL	SC-Leucine
pWS2125	Recombination-mediated PCR-directed plasmid construction in yeast (BY4741)	Restriction enzyme digested pWS2101	PCR product encoding C-terminus of *RHO1* with CXXX sequence replaced with a *BamHI* site	SC-Leucine
pWS2326	Recombination-mediated PCR-directed plasmid construction in yeast (BY4741)	Restriction enzyme digested pWS1934	PCR product encoding *CDC43*	SC-Leucine
pWS2327	Recombination-mediated PCR-directed plasmid construction in yeast (BY4741)	Restriction enzyme digested pWS1885	PCR product encoding *RAM2*	SC-Leucine
pWS2350	Ligation	Restriction enzyme digested pWS2327	Restriction enzyme digested pRS413	Ampicillin
